# Impact of CT-Defined Sarcopenia on Clinical Outcomes in Elderly Trauma Patients: A Retrospective Korean Cohort Study

**DOI:** 10.3390/healthcare13182321

**Published:** 2025-09-16

**Authors:** Juhong Park, Yesung Oh, Songhee Kwon, Jihyun Lee, Mihyang Kim, Donghwan Choi, Junsik Kwon

**Affiliations:** 1 Division of Trauma Surgery, Department of Surgery, Ajou University School of Medicine, Suwon 16499, Republic of Korea; doginzoo@aumc.ac.kr (J.P.); claptonc@naver.com (D.C.); 2 Department of Food Service and Clinical Nutrition, Ajou University Hospital, Suwon 16499, Republic of Korea; yesung@aumc.ac.kr (Y.O.); 111783@aumc.ac.kr (S.K.); futurist21c@aumc.ac.kr (J.L.); a24withu@aumc.ac.kr (M.K.)

**Keywords:** sarcopenia, trauma, elderly patients, computed tomography

## Abstract

**Background/Objectives:** Sarcopenia, the age-related decline in skeletal muscle mass and function, is increasingly recognized as an important prognostic factor among elderly patients. This study aimed to evaluate whether computed tomography (CT)-defined sarcopenia independently predicts short-term mortality in elderly Korean trauma patients. **Methods:** We retrospectively analyzed 722 patients aged ≥65 years admitted to a Korean Level I trauma center between January 2020 and December 2021. Sarcopenia was defined as the lowest sex-specific quartile of skeletal muscle index (SMI) measured at the third lumbar vertebra (L3) within 7 days of admission. Demographics, injury severity, and outcome variables were compared between groups. Kaplan–Meier survival analysis with a 24 h landmark and multivariable Cox regression were applied to identify independent predictors of 30-day mortality. **Results:** Among 722 patients, 181 (25.1%) were sarcopenic. They were older and had lower body mass index and serum albumin yet showed lower Injury Severity Score (ISS) at presentation. Despite this, in-hospital mortality was higher in sarcopenic patients (15.5% vs. 9.8%, *p* = 0.036), while 24 h mortality did not differ (4.4% vs. 3.7%, *p* = 0.663). Landmark analysis starting at 24 h demonstrated significantly worse 30-day survival in the sarcopenia group (log-rank *p* = 0.028). Multivariable Cox regression confirmed sarcopenia as an independent predictor of 30-day mortality (HR, 2.36; 95% CI, 1.07–5.23; *p* = 0.034), along with higher ISS and lower Glasgow Coma Scale (GCS) scores. **Conclusions:** CT-defined sarcopenia at the L3 level independently predicts 30-day mortality in elderly trauma patients and may support early risk stratification.

## 1. Introduction

Trauma is a leading cause of death worldwide among individuals under 40 years and remains a major health concern in both high- and low-income countries [1]. Although immediate mortality may result from catastrophic injuries [2], older trauma patients show disproportionately high mortality even after adjusting for injury severity. This excess risk is mainly attributed to pre-existing comorbidities and diminished physiological reserve, often termed frailty [3,4], which has been recognized as a key determinant of poor outcomes in elderly trauma patients [5,6].

Sarcopenia, the age-related decline in skeletal muscle mass and function [7,8], represents a biological substrate of frailty [9] and is strongly associated with adverse outcomes in older adults, including increased mortality, functional decline, and higher rates of hospitalization [10]. The computed tomography (CT)-based skeletal muscle index (SMI), calculated by normalizing cross-sectional skeletal muscle area (SMA) at the third lumbar vertebra (L3) to height squared, has emerged as a reliable method for assessing sarcopenia [11,12,13]. Prior work has shown that L3 muscle mass correlates with grip strength and gait speed, supporting its clinical utility as a surrogate marker of functional status [14,15]. In trauma populations, abdominal CT is frequently performed during the initial evaluation, making it a practical modality for assessing skeletal muscle mass.

Several studies in Western populations have demonstrated that CT-defined sarcopenia predicts poor outcomes after trauma [16,17]. These studies consistently confirmed sarcopenia as an independent predictor of mortality. However, evidence from Asian populations remains limited in the trauma field, despite well-documented racial differences in muscle mass between Asians and Westerners [18]. Therefore, this study aimed to examine the association between CT-defined sarcopenia and clinical outcomes, particularly 30-day mortality, major complications, hospital length of stay (LOS) in elderly trauma patients in Korea, hypothesizing that sarcopenia would be independently associated with increased mortality and adverse outcomes.

## 2. Materials and Methods

### 2.1. Patient Selection

**Figure 1:**
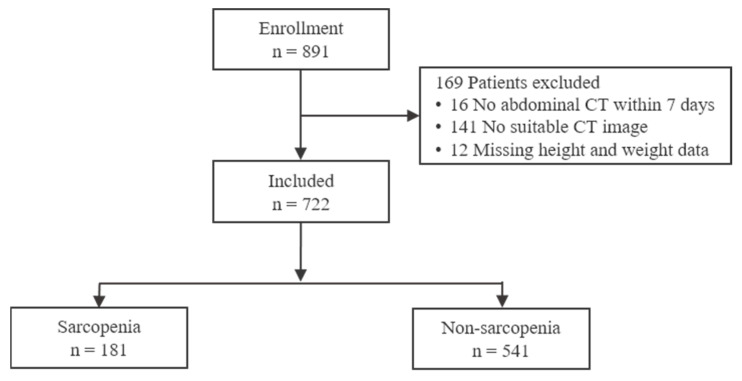
Flowchart of patient inclusion and exclusion.

This retrospective analysis utilized data extracted from the Korean Trauma Data Bank (KTDB). We identified 891 patients aged 65 years or older who had been admitted to a designated Level I trauma center between January 2020 and December 2021. Patients were included if they underwent abdominal CT within one week of admission. Patients without adequate L3-level CT images or relevant clinical data were excluded (Figure 1).

Ethical approval was obtained from the Institutional Review Board of Ajou University Hospital (AJOUIRB-DB-2023-102), and the requirement for informed consent was waived due to the retrospective nature of the study. This study was conducted and reported in accordance with the STROBE (Strengthening the Reporting of Observational Studies in Epidemiology) guidelines, and the completed checklist is provided in the Supplementary Materials.

### 2.2. Data Collection

Patient information was obtained from electronic medical records, including demographic characteristics such as age, sex, weight, and body mass index (BMI), along with selected clinical variables. The severity of traumatic injuries was quantified using the Abbreviated Injury Scale (AIS) and the Injury Severity Score (ISS), together with vital signs and laboratory findings at emergency department arrival. Clinical outcomes included in-hospital mortality, total transfusion volume, duration of mechanical ventilation, overall hospital stay, intensive care unit (ICU) admission duration, and cumulative hospitalization expenses.

### 2.3. SMI Calculation

**Figure 2:**
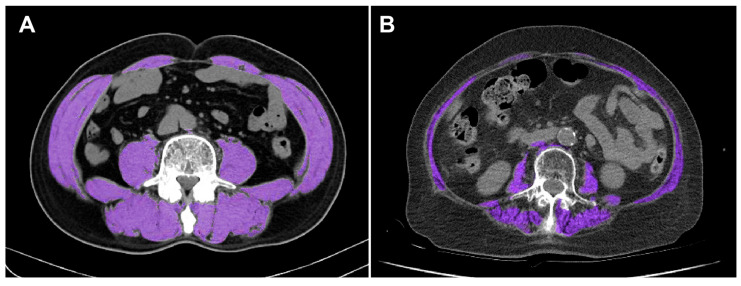
Example of CT-based evaluation of skeletal muscle at the L3 level in two representative patients: (**A**) non-sarcopenia and (**B**) sarcopenia. Skeletal muscle areas are highlighted in purple.

Abdominal CT scans were retrospectively reviewed to measure SMA. Images were acquired using a 64-slice Siemens SOMATOM Definition Flash scanner (Siemens Healthineers, Erlangen, Germany). Cross-sectional images at the level of L3 were selected, and skeletal muscle was defined as tissue with attenuation values between −30 and 150 Hounsfield units. Body composition parameters, including SMA, subcutaneous fat area, and visceral fat area, were quantified with the AsanJ-Morphometry™ software (Asan Medical Center, Seoul, Republic of Korea) [19], which was developed on the ImageJ platform (National Institutes of Health, Bethesda, MD, USA) [20]. The measured SMA at the L3 level included the psoas, quadratus lumborum, transversus abdominis, internal and external obliques, rectus abdominis, and erector spinae muscles, as shown in Figure 2.

The SMI was calculated by dividing SMA (cm^2^) by height squared (m^2^):

$$SMI=\frac{SMA\;\left(cm^{2}\right)}{height^{2}\;\left(m^{2}\right)}$$

### 2.4. Definition of Sarcopenia in This Study

Consensus definitions of sarcopenia require both reduced muscle mass and impaired muscle strength or physical performance [7,18]. In acute trauma settings, however, functional assessments are rarely available, and no universally accepted cutoff values specific to trauma populations, especially in Asia, have been established. In this study, sarcopenia was identified when SMI values fell within the lowest quartile of the sex-specific distribution, consistent with prior distribution-based approaches [21,22]. Accordingly, participants were stratified into sarcopenic and non-sarcopenic groups using these threshold values, and their clinical outcomes were subsequently compared. Based on our cohort, the derived cutoff points were 36.37 cm^2^/m^2^ for men and 27.86 cm^2^/m^2^ for women, and these values were applied to define the two groups.

### 2.5. Statistical Analysis

All statistical analyses were performed using SPSS software, version 25.0 (IBM Corp., Armonk, NY, USA) and R software, version 4.3.1 (R Foundation for Statistical Computing, Vienna, Austria).

First, baseline demographic and clinical characteristics were compared between sarcopenic and non-sarcopenic patients (Table 1). Continuous variables were examined for normality using the Kolmogorov–Smirnov test. Normally distributed variables were presented as means with standard deviations and compared using Student’s *t*-test. Non-normally distributed variables were summarized as medians with interquartile ranges and analyzed with the Mann–Whitney U test. Categorical variables were expressed as counts with percentages and assessed using the chi-square or Fisher’s exact test, as appropriate.

Second, clinical outcomes, including in-hospital mortality, 24 h mortality, hospital LOS, total hospital costs, and major complications, were compared between groups to evaluate associations with sarcopenia (Table 2 and Table 3). Hospital costs, originally recorded in Korean Won (KRW), were converted to United States Dollars (USD) using an approximate exchange rate of 1 USD = 1200 KRW.

**Figure 3:**
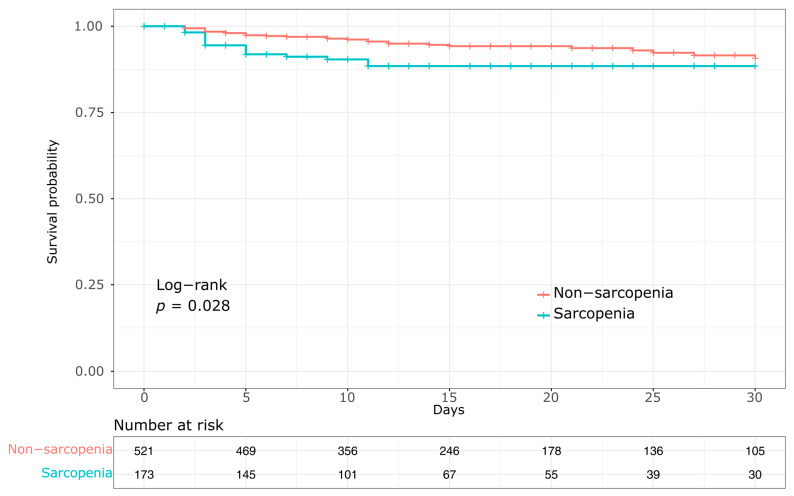
Landmark Kaplan–Meier survival curves starting at 24 h after admission and continuing through 30 days, comparing sarcopenia and non-sarcopenia groups.

Third, survival analysis was conducted using a landmark Kaplan–Meier approach to illustrate differences in mortality beyond the first 24 h after admission. Patients who died within 24 h were excluded from this analysis, as early deaths are primarily attributable to unsurvivable injuries or massive hemorrhage and are unlikely to be influenced by sarcopenia. Survival curves were constructed up to 30 days, and patients who were discharged alive prior to death were censored at the time of discharge. Group survival curves were compared using the log-rank test (Figure 3).

Finally, to identify independent predictors of mortality, we performed a Cox proportional hazards regression analysis. Variables with a *p* < 0.10 in univariate analyses were considered candidates for the multivariable model, and clinically relevant variables such as age and sex were included regardless of their univariate significance (Table 4).

All statistical tests were two-sided, and *p* < 0.05 was considered statistically significant.

## 3. Results

### 3.1. Patient Demographics and Characteristics

From January 2020 to December 2021, 891 patients aged ≥65 years were registered in the KTDB. A total of 169 patients were excluded for the following reasons: absence of abdominal CT within the first week of admission (n = 16), inadequate L3-level CT images caused by artifacts or temporary abdominal closure (n = 141), or missing anthropometric data such as height or weight (n = 12). Accordingly, 722 patients (486 males and 236 females) comprised the final study cohort.

The lowest sex-specific SMI quartiles were 36.37 cm^2^/m^2^ for men and 27.86 cm^2^/m^2^ for women; 181 patients (25.1% overall; 122 males and 59 females) below these thresholds were classified as sarcopenic, and the remaining 541 patients were assigned to the non-sarcopenia group.

Baseline characteristics are presented in Table 1. Patients in the sarcopenia group were significantly older (median 77 vs. 72 years, *p* < 0.001), with a higher proportion aged ≥75 years (61.9% vs. 36.0%, *p* < 0.001). They also had lower body weight, BMI, serum albumin, and hemoglobin levels at admission (all *p* < 0.05). The sarcopenia group exhibited a lower median ISS and a smaller proportion of patients with ISS ≥ 16. Severe thoracic and abdominal injuries (AIS ≥ 3) were more frequently observed in the non-sarcopenia group (both *p* < 0.05).

### 3.2. Clinical Outcomes and Complications

As shown in Table 2, the sarcopenia group had a significantly shorter overall hospital LOS compared with the non-sarcopenia group (12 [6,7,8,9,10,11,12,13,14,15,16,17,18,19,20,21,22] vs. 14 [8,9,10,11,12,13,14,15,16,17,18,19,20,21,22,23,24,25,26] days, *p* = 0.026). No significant differences were observed between the two groups in ICU LOS, use of mechanical ventilation, ventilator days, or transfusion-related parameters, including the proportion of patients receiving red blood cell (RBC) transfusion, transfusion amount within 24 h, and the frequency of massive transfusion. Although the median total in-hospital costs were lower in the sarcopenia group (2.87 × 10^4^ USD vs. 3.62 × 10^4^ USD), the difference did not reach statistical significance (*p* = 0.053).

Regarding in-hospital complications (Table 3), there were no statistically significant differences between the two groups across all evaluated complications, including sepsis, catheter-associated infections, acute kidney injury, ventilator-associated pneumonia, acute respiratory distress syndrome, cardiac arrest, pulmonary embolism, stroke, or myocardial infarction.

### 3.3. Mortality

Overall, 81 patients (11.2%) died during hospitalization. In-hospital mortality was significantly higher in the sarcopenia group compared with the non-sarcopenia group (15.5% vs. 9.8%, *p* = 0.036). Early mortality within 24 h occurred in 28 patients (34.6% of all deaths), and the proportion of early deaths did not differ significantly between the two groups (4.4% vs. 3.7%, *p* = 0.663). Landmark Kaplan–Meier curves starting at 24 h and continuing through 30 days demonstrated significantly reduced survival in the sarcopenia group compared with the non-sarcopenia group (log-rank *p* = 0.028) (Figure 3).

### 3.4. Cox Regression Analysis

To further evaluate the independent effect of sarcopenia on survival, a Cox proportional hazards regression analysis was performed in 694 patients who survived beyond the first 24 h (Table 4). In the univariate analysis, sarcopenia (HR, 1.93; 95% CI, 1.06–3.51; *p* = 0.032), SBP ≤ 90 mmHg (HR, 1.01; 95% CI, 1.00–1.02; *p* = 0.022), ISS (HR, 1.05; 95% CI, 1.03–1.07; *p* < 0.001), lower GCS (HR, 0.73; 95% CI, 0.68–0.78; *p* < 0.001), and albumin level (HR, 0.62; 95% CI, 0.39–0.97; *p* = 0.038) were significantly associated with 30-day mortality.

In the multivariable model, sarcopenia remained an independent predictor of 30-day mortality (HR, 2.36; 95% CI, 1.07–5.23; *p* = 0.034), along with higher ISS (HR, 1.06; 95% CI, 1.02–1.09; *p* = 0.002) and lower GCS (HR, 0.74; 95% CI, 0.68–0.80; *p* < 0.001).

## 4. Discussion

This retrospective cohort study showed that lower skeletal muscle mass on CT—quantified by the L3 SMI was associated with higher 30-day mortality in elderly Korean trauma patients, even after adjustment for injury severity and other covariates (HR, 2.36; 95% CI, 1.07–5.23; *p* = 0.034). Early mortality within 24 h was similar between sarcopenic and non-sarcopenic patients (4.4% vs. 3.7%; *p* = 0.663), suggesting that sarcopenia likely exerts only a limited influence on hyperacute-phase deaths, which are typically driven by massive hemorrhage or unsurvivable injuries. Landmark Kaplan–Meier survival curves, starting at 24 h after admission to exclude early deaths, demonstrated significantly lower 30-day survival in the sarcopenia group (log-rank *p* = 0.028).

These findings are consistent with a systematic review and meta-analysis of 10 retrospective studies among trauma populations (n = 2867), in which CT-assessed sarcopenia was associated with higher in-hospital mortality (RR, 1.96; 95% CI, 1.30–2.94) and 30-day mortality (RR, 1.60; 95% CI, 1.21–2.13) [23]. Likewise, in an elderly trauma cohort (n = 204), central sarcopenia defined by the psoas-to-lumbar vertebral index (PLVI ≤ 0.53) was associated with higher in-hospital mortality (33% vs. 13%), remaining significant after adjustment (adjusted OR, 3.38; 95% CI, 1.47–9.73; *p* = 0.006) [24].

Considering why sarcopenia is linked to poorer outcomes, several biologically plausible pathways have been proposed: lower skeletal muscle mass may reflect reduced physiologic and metabolic reserve, blunted immune–inflammatory modulation via myokines, and respiratory muscle weakness, each of which can hinder recovery from shock, infection, and surgery [25,26]. Sarcopenia also commonly coexists with malnutrition and reduced functional capacity, potentially delaying mobilization, increasing vulnerability to complications, and limiting responsiveness to critical care [27,28]. These factors are not fully captured by standard anatomical injury measures.

The sarcopenia group was generally older, with a larger proportion aged ≥75 years, consistent with longitudinal evidence demonstrating an age-related decline in skeletal muscle mass and quality [29]. Aging is a major risk factor for sarcopenia reflecting cumulative physiological decline with advancing age [30]. In our multivariable analysis, age itself also remained independently associated with death, reinforcing the strong contribution of advanced age to poor outcomes. These observations underscore the importance of early and objective evaluation of muscle status in elderly trauma patients, which can inform timely nutritional assessment and rehabilitation strategies to prevent further muscle loss.

Importantly, despite having a lower mean ISS, the sarcopenia group experienced higher mortality. Previous studies in geriatric trauma patients have also shown that ISS alone does not fully capture the impact of comorbidities and frailty; for instance, elderly patients with rib fractures demonstrate nearly double the mortality of younger patients despite lower ISS and higher GCS [31], and the World Society of Emergency Surgery (WSES) guideline similarly identifies age ≥ 65 years as an independent risk factor for trauma mortality even after adjusting for ISS, with a 2.4- to 5.6-fold increased risk of death [32]. These findings underscore that anatomical scores such as ISS are insufficient to reflect the vulnerability of elderly trauma patients. Given that sarcopenia represents a central component of frailty and is strongly age-related, the excess mortality observed in sarcopenic patients with lower ISS may, at least in part, reflect this frailty-driven vulnerability. This paradox highlights the limitation of ISS as an anatomical score alone and emphasizes the need to incorporate host factors such as sarcopenia into trauma prognostication.

Hospital LOS was shorter in the sarcopenia group (12 [6,7,8,9,10,11,12,13,14,15,16,17,18,19,20,21,22] vs. 16 [8,9,10,11,12,13,14,15,16,17,18,19,20,21,22,23,24,25] days; *p* = 0.026). Total in-hospital costs were numerically lower but did not reach statistical significance (*p* = 0.053). Although sarcopenic patients presented with a lower ISS, which might have contributed to their shorter LOS and lower costs, this factor alone cannot fully explain the findings. Shorter stays may partly reflect higher intermediate-term mortality because patients who die sooner cannot accumulate LOS, a well-recognized issue when death competes with LOS metrics [33]. Consistent with this interpretation, in our 24 h landmark analysis, 30-day survival was significantly lower in the sarcopenia group, and the multivariable Cox model confirmed a higher hazard of death.

Complication rates did not differ significantly between groups in our cohort, consistent with prior studies. A CT-based cohort using an L4 psoas-to-vertebral area index reported an adjusted OR of 1.08 (95% CI, 0.52–2.21) for any complication [24], and an ICU-admitted elderly trauma cohort observed similar rates (14.9–20.8%; *p* = 0.85) [34]. In our data, the low frequency of individual complications limited statistical power to detect modest differences, and earlier mortality reduced the time at risk for recording complications, potentially underestimating between-group contrasts when death was a competing event.

This study has several strengths. It focuses on an Asian elderly trauma cohort, an underrepresented population in CT-based sarcopenia research whose body-composition profiles may differ from those of Western cohorts. In addition, we applied careful analytical strategies, including a 24 h landmark approach to reduce bias from nonsurvivable injuries and a multivariable model that accounted for key demographic and clinical factors. These approaches strengthen the validity of our findings and suggest that sarcopenia could serve as a prognostic marker in elderly trauma patients.

This study has several limitations. First, its retrospective, single-center design limits generalizability and raises the possibility of selection bias, including exclusion of patients without adequate admission CT or complete anthropometrics. Second, sarcopenia was defined by a single cross-sectional measure of muscle quantity at admission; muscle quality, including CT radiodensity/myosteatosis, and strength were not assessed. Prior studies have shown that muscle strength and quality decline more rapidly with age than muscle mass and that functional measures may better predict outcomes than muscle mass alone [35,36]. Additionally, we did not evaluate within-hospital changes in muscle quantity. Serial CT-based studies have reported measurable post-admission skeletal muscle loss, and CT-documented in-hospital loss has been associated with poorer outcomes in severe trauma [37]. Third, we lacked comprehensive nutritional and pre-injury functional data, and unmeasured factors (e.g., comorbidity burden, broader frailty domains) may have confounded the results. Finally, the 24 h landmark approach mitigates but does not eliminate survivorship bias; we did not evaluate time-varying hazards or alternative survival summaries, and outcomes were only tracked to 30 days.

Notwithstanding these limitations, our findings suggest that CT-based skeletal muscle assessment may serve as a practical and objective tool for early risk stratification in elderly trauma patients. Because abdominal CT is frequently obtained during initial trauma evaluation, L3 SMI can be calculated from existing images at no additional scan, radiation, or cost, facilitating potential integration into clinical practice.

Prospective multicenter studies are now needed to measure muscle quantity and quality at admission using CT to standardize assessments of handgrip strength, gait speed, respiratory muscle performance, and nutritional status, and to obtain follow-up imaging to capture in-hospital muscle loss. Longer-term follow-up should be used to derive and externally validate an Asian-specific risk model that integrates these measures with clinical variables such as age, sex, ISS, and GCS, including calibration of population-appropriate cut points.

## 5. Conclusions

In conclusion, our findings suggest that CT-defined sarcopenia is independently associated with 30-day mortality in elderly trauma patients and may serve as a prognostic marker. Given the widespread use of abdominal CT in the assessment of trauma patients, early identification of sarcopenia through CT-based skeletal muscle mass measurement could help clinicians develop targeted treatment strategies to improve patient outcomes.

## Figures and Tables

**Table 1:** Patient Demographics and Characteristics.

Variable	Sarcopenia (n = 181)	Non-Sarcopenia (n = 541)	*p*-Value
Age, years	77 [72–82]	72 [67–78]	<0.001
Age ≥ 75 years, n (%)	112 (61.9)	195 (36.0)	<0.001
Sex, n (%)			0.976
Male	122 (67.4)	364 (67.3)	
Female	59 (32.6)	177 (32.7)	
Weight, kg	59.6 ± 11.4	64.3 ± 10.9	<0.001
BMI, kg/m^2^	22.0 ± 3.46	24.1 ± 3.06	<0.001
Blunt injury, n (%)	178 (98.3)	523 (96.7)	0.247
Mechanism of injury, n (%)			0.037
Fall	97 (53.6)	225 (41.6)	
Motor vehicle accident	17 (9.4)	84 (15.5)	
Motorcycle accident	16 (8.8)	49 (9.1)	
Bicycle accident	5 (2.8)	37 (6.8)	
Stabbing	3 (1.7)	15 (2.8)	
Pedestrian	29 (16.0)	97 (17.9)	
Struck by	4 (2.2)	15 (2.8)	
Other Mechanisms	1 (0.6)	6 (1.1)	
Unknown	9 (5.0)	13 (2.4)	
SBP, mmHg	143 [121–158]	143 [124–161]	0.997
SBP ≤ 90 mmHg, n (%)	9 (5.0)	22 (4.1)	0.603
GCS	15 [14,15]	15 [14,15]	0.953
ISS	18 [10–26]	19 [14–29]	0.013
ISS ≥ 16, n (%)	104 (57.5)	358 (66.2)	0.034
AIS Head ≥ 3, n (%)	21 (11.6)	88 (16.3)	0.129
AIS Thorax ≥ 3, n (%)	94 (51.9)	367 (67.8)	<0.001
AIS Abdomen ≥ 3, n (%)	7 (3.9)	60 (11.1)	0.004
Albumin, g/dL	3.8 [3.4–4.1]	3.9 [3.6–4.2]	0.010
Hemoglobin, g/dL	11.7 [10.1–12.9]	12.4 [10.9–13.4]	<0.001
SMA, cm^2^	83.8 [65.9–97.0]	114.0 [89.5–127.1]	<0.001
SMI, cm^2^/m^2^	29.47 ± 5.05	41.20 ± 7.33	<0.001

Values are presented as median [IQR], mean ± standard deviation, or number (%). BMI, body mass index; SBP, systolic blood pressure; GCS, Glasgow Coma Scale; ISS, Injury Severity Score; AIS, Abbreviated Injury Scale; SMA, skeletal muscle area; SMI, skeletal muscle index.

**Table 2:** Clinical outcomes.

Outcome	Sarcopenia (n = 181)	Non-Sarcopenia (n = 541)	*p*-Value
24 h mortality, n (%)	8 (4.4)	20 (3.7)	0.663
In-hospital mortality, n (%)	28 (15.5)	53 (9.8)	0.036
Hospital LOS	12 [6–22]	14 [8–26]	0.026
ICU LOS	6.5 [3–20]	6.5 [2–17]	0.654
Mechanical ventilation, n (%)	58 (32.0)	192 (35.5)	0.399
Ventilator days	5 [4–8]	3 [2–7]	0.695
RBC transfusion, n (%)	11 (6.1)	44 (8.1%)	0.367
RBC transfusion within 24 h, unit	13.5 [8–15]	12 [7–16]	1.000
Massive transfusion * n (%)	11 (6.1)	44 (8.1)	0.367
Hospital cost, USD ** × 10^4^ $	2.87 [2.29–4.38]	3.62 [1.50–5.87]	0.053

Values are presented as median [IQR] or number (%). LOS, length of stay; ICU, intensive care unit; RBC, red blood cell. * Transfusion of ≥10 units of RBC within 24 h. ** Hospital costs were converted from KRW to USD at an approximate exchange rate of 1 USD = 1200 KRW.

**Table 3:** In-hospital complications in sarcopenic and non-sarcopenic patients.

Complication (n, %)	Sarcopenia (n = 181)	Non-Sarcopenia (n = 541)	*p*-Value
Sepsis	3 (1.7)	16 (3.0)	0.431
CAUTI	3 (1.7)	13 (2.4)	0.772
Deep or Organ SSI	0	3 (0.6)	0.577
CLABSI	0	3 (0.6)	0.577
Acute kidney injury	5 (2.8)	29 (5.4)	0.153
VAP	8 (4.4)	33 (6.1)	0.398
ARDS	4 (2.2)	8 (1.5)	0.508
Cardiac arrest	6 (3.3)	14 (2.6)	0.606
Pulmonary embolism	0	4 (0.7)	0.577
Stroke	2 (1.1)	7 (1.3)	1.000
Myocardial infarction	0	2 (0.4)	1.000

CAUTI, catheter-associated urinary tract infection; SSI, surgical site infection; CLABSI, central line-associated bloodstream infection; VAP, ventilator-associated pneumonia; ARDS, acute respiratory distress syndrome.

**Table 4:** Cox proportional hazards regression analysis for 30-day mortality in patients surviving beyond 24 h.

Variable	Univariate Analysis		Multivariable Analysis	
	HR (95% CI)	*p*-Value	HR (95% CI)	*p*-Value
Age	1.03 (0.99–1.07)	0.157	1.00 (0.95–1.06)	0.927
Male	1.06 (0.57–2.00)	0.847	0.68 (0.32–1.42)	0.301
Sarcopenia	1.93 (1.06–3.51)	0.032	2.36 (1.07–5.23)	0.034
SBP ≤ 90	1.01 (1.00–1.02)	0.022	1.37 (0.34–5.57)	0.663
ISS	1.05 (1.03–1.07)	<0.001	1.06 (1.02–1.09)	0.002
GCS	0.73 (0.68–0.78)	<0.001	0.74 (0.68–0.80)	<0.001
Albumin	0.62 (0.39–0.97)	0.038	1.02 (0.52–1.99)	0.955

SBP, systolic blood pressure; ISS, Injury Severity Score; GCS, Glasgow Coma Scale.

## Data Availability

The data presented in this study are available on request from the corresponding author. The data are not publicly available due to privacy and ethical restrictions.

## Supplementary Materials

The following supporting information can be downloaded at: https://www.mdpi.com/article/10.3390/healthcare13182321/s1, File S1: STROBE Statement—Checklist of items that should be included in reports of cohort studies.
